# Observation vs. interaction in the recognition of human-like movements

**DOI:** 10.3389/frobt.2023.1112986

**Published:** 2023-04-10

**Authors:** Giovanni Mignone, Antonio Parziale, Enrico Ferrentino, Angelo Marcelli, Pasquale Chiacchio

**Affiliations:** Department of Information Engineering, Electrical Engineering, and Applied Mathematics, University of Salerno, Fisciano, Italy

**Keywords:** collaborative robots, human-robot collaboration, human-robot interaction, robot acceptance, turing test

## Abstract

A crucial aspect in human-robot collaboration is the robot acceptance by human co-workers. Based on previous experiences of interaction with their fellow beings, humans are able to recognize natural movements of their companions and associate them with the concepts of trust and acceptance. Throughout this process, the judgment is influenced by several percepts, first of all the visual similarity to the companion, which triggers a process of self-identification. When the companion is a robot, the lack of these percepts challenges such a self-identification process, unavoidably lowering the level of acceptance. Hence, while, on the one hand, the robotics industry moves towards manufacturing robots that visually resemble humans, on the other hand, a question is still open on whether the acceptance of robots can be increased by virtue of the movements they exhibit, regardless of their exterior aspect. In order to contribute to answering this question, this paper presents two experimental setups for Turing tests, where an artificial agent performs human-recorded and artificial movements, and a human subject is to judge the human likeness of the movement in two different circumstances: by observing the movement replicated on a screen and by physically interacting with a robot executing the movements. The results reveal that humans are more likely to recognize human movements through interaction than observation, and that, under the interaction condition, artificial movements can be designed to resemble human ones for future robots to be more easily accepted by human co-workers.

## 1 Introduction

The human acceptance of robots’ behavior is key to designing suitable human-robot interaction schemes and related control algorithms (Kim et al., 2012). From the human standpoint, robots should mimic human motor functions. Possible strategies are to design robots with anthropomorphic aspects (Averta et al., 2020) and/or to assign them a specific behavior, concerning movement, speech and visual/facial gestures, shaping their interaction with humans (Kelley et al., 2010).

Focusing on the latter, one of the main differences between human and classical robotic arm movements is observed in their velocity features. The Kinematic Theory of rapid human movements (Plamondon, 1995), and its consequent sigma-lognormal model (O’Reilly and Plamondon, 2009), suggest that complex human movements are the result of the time superimposition of elementary movements, each of which is commanded by the central nervous system and exhibits a lognormal velocity profile. Throughout the years, the sigma-lognormal model has proved to be effective at reproducing human-like movements in 2D (Ferrer et al., 2020; Parziale et al., 2020; Laurent et al., 2022) and 3D conditions (Fischer et al., 2020). On the other side, typical robotic/artificial velocity profiles are constant or trapezoidal (Kavraki and LaValle, 2016).

Given this difference, questions arise on whether humans are able to discriminate between human and artificial movements and whether the modalities by which humans perceive the movement can affect the judgment about its human likeness. Chamberlain et al. (2022) designed experiments consisting in the observation of videos and claimed that human-like drawing movements with bell-shaped velocity profiles are perceived as more natural and pleasant than movements with a uniform profile. On the contrary, Quintana et al. (2022) showed how humans do not have a clear preference between movements at constant and lognormal speed, both when watching videos of a robotic arm executing some trajectories in space and when interacting with the robot by tracking its tip with their finger.

Some works focused on the design of new control strategies able to provide a robot with more comfortable movements during human-robot interaction tasks. Tamantini et al. (2022) presented a patient-tailored control architecture for upper-limb robot-aided orthopedic rehabilitation. They designed a learning-by-demonstration-based approach using Dynamic Movement Primitives (DMP). The proposed controller is capable of adapting the rehabilitation workspace and the assistance forces according to the patient’s performance. Similarly, Lauretti et al. (2019) proposed a new formulation of DMP that endows anthropomorphic robots with the capability of performing movements similar to the human demonstrator both in the joint and Cartesian space and avoiding obstacles. The proposed approach was compared with a literature method based on Cartesian DMP and (IK). The questionnaire results showed that the users prefer the anthropomorphic motion planned through the proposed approach with respect to the non-anthropomorphic one planned by means of Cartesian DMP and IK.

Our contribution is to investigate to which extent the modalities by which humans perceive motion affect their judgment about the human likeness of movements. Our results show that the physical interaction with the robot simplifies the detection of human movements if compared to the mere observation of the same movements. In our view, this supports the thesis that, depending on the task, robots can be provided with different degrees of human likeness, in view of acceptance. Also, in the same view, not all artificial trajectories are the same, but some are more frequently perceived as human than others. This paves the way to the design of robots resembling human behavior and, therefore, is expected to improve robot acceptance in human-robot collaboration.

The remainder of the paper is organized as follows: Section 2 describes two Turing tests, each featuring a different experimental setup; Section 3 presents the results of such experiments and Section 4 discusses the results and provides concluding remarks.

## 2 Materials and methods

With the focus on robot acceptance, we designed our experiments to excite the visual and the proprioceptive systems separately, and to analyze which of them is more effective in the view of human-robot collaboration. The experiments are shaped in the form of Turing tests: the stimulus provided to a human subject is a handwriting motion, and the subject is required to decide whether it is produced by a human or by an artificial agent.

### 2.1 Participants

The participants were recruited among members of the Natural Computation and Robotics Laboratories and students attending the M.Eng. degrees at our department. All the participants accepted to participate on a voluntary basis, and formally expressed their consent to participate by reading and signing a consensus form. The participants were divided in two disjoint groups, referred to as G1 and G2. The nine subjects of G1 provided the human trajectories, while the 36 of G2 participated in the tests. None of the subjects of G2 had previous experience of physical interaction with a robot. Details about the participants included in G1 and G2 are reported in the Supplementary Table S1.

### 2.2 Generation of trajectories

In general, the equipment adopted to record movements and the motor task that is investigated characterize studies on human movements. Recording systems (motion tracker, tablet, smartwatch, etc.) and motor tasks (gait, reaching movements, handwriting, etc.) are selected according to the final application or the particular aspect of the movements to be investigated. Pen-tip movements during signing, drawing or writing acquired with graphic tablets and smartpads have been largely adopted (Diaz et al., 2019; Parziale et al., 2021; Cilia et al., 2022), but video recording of gait as well as food manipulation during feeding have also been suggested (Bhattacharjee et al., 2019; Kumar et al., 2021).

**FIGURE 1:**
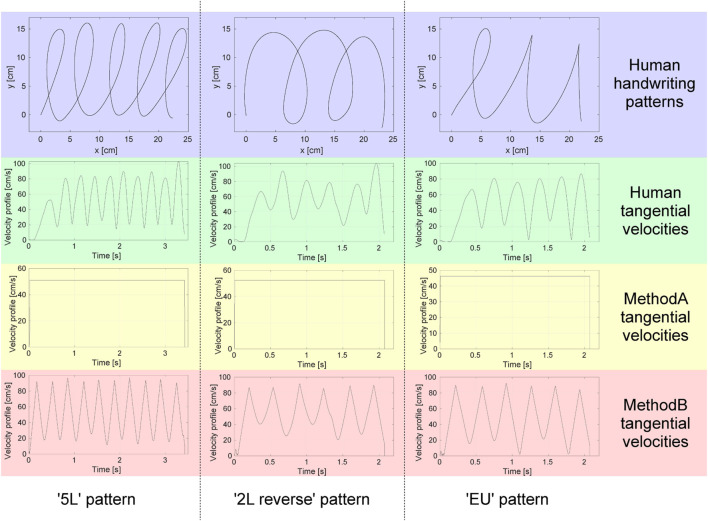
Human handwriting patterns and time laws.

In our work, the motor task we opted for is the drawing of the simple shapes illustrated in the top panel of Figure 1, because they do not trigger the processing of semantic information in the subjects involved in the experiments. The advantage of adopting these shapes instead of characters, words, signatures or other goal-driven actions, is that of not activating cortex regions devoted to integrating semantic information, as it would happen if the task had a semantic content (Harrington et al., 2009), thus avoiding cognitive biases that could affect the experimental results. Notwithstanding, the tasks are not simple from a motor perspective, in that their execution requires the proper synchronization of many elementary movements.

**FIGURE 2:**
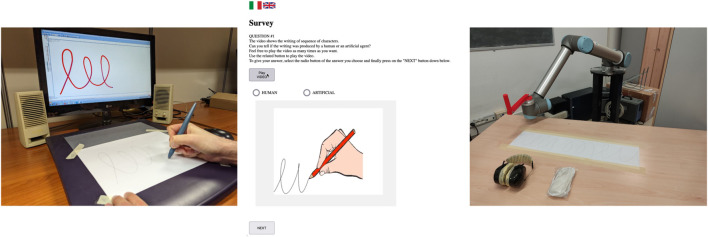
Acquisition setup (left), observation experiment setup (center), interaction experiment setup (right).

The subjects of G1 were requested to draw the proposed shapes with a ballpoint pen on a sheet of paper placed on an ink-and-paper WACOM Intuos 2 digitizing tablet with a 100 Hz sampling rate. The motion of the ballpoint pen over the 2D plane of the tablet was recorded through the software MovAlyzeR^®^v6.1 (Teulings, 2021). The subjects were instructed to draw 10 times each pattern so as to occupy as much as possible of the A4 surface of the sheet. They were free to write at their own pace and only the on-paper movements were recorded. The acquisition setup is reported in Figure 2.

From the recorded human trajectories, we extracted the geometrical features, i.e., the path in planar coordinates, and assigned each of them two different time laws from robotics literature to move along, referred to as MethodA (Kavraki and LaValle, 2016) and MethodB (Slotine and Yang, 1989). In particular, MethodA assumes that the velocity is uniform, while MethodB assumes that the velocity depends on curvature, according to a time-optimal profile, as in the case of human movements. Thus, for each pattern, there are three different execution modalities, which we term *classes*, one human and two artificial, denoted in the sequel as *Human*, *MethodA* and *MethodB*. We use the term *Human* to denote trajectories that, although executed by an artificial agent, are not obtained by any model, but are a mere replication of the human movements acquired through the graphic tablet. For more information about MethodB trajectories, refer to the Supplementary Material.

### 2.3 Observation experiment

In this experiment only visual stimuli are provided to the subjects for making a decision. To this purpose, we generated animations of the trajectories in compliance with human and artificial time laws. Based on the assumption that context information have an effect on the judgment of human likeness, we stimulated the self-identification with the execution of a handwriting task by including an icon of a human hand holding a pen, and generating the animated trajectory as the hand moves, so as to simulate a pen releasing ink on a sheet. This represents the major difference with respect to the experiments reported by (Chamberlain et al., 2022) and (Quintana et al., 2022), as we purged videos from the visual clues that could divert the attention of the individual from the movement (such as a robotic manipulator structure), and include elements to help the human subject in the self-identification process.

The visual stimuli are presented as a sequence of 10 instances of the aforementioned patterns, generated by randomly selecting three human trajectories, one for each pattern, and, for each of them, the corresponding trajectories generated by MethodA and MethodB, respectively, plus one more artificial trajectory generated by MethodB. During the test, one trajectory animation at a time is presented on a screen, and the participant is requested to decide if the movements in the video are produced by a human or by an artificial agent. The test is designed in such a way to avoid a participant skipping or pausing a video, going back to already answered questions or repeating the entire survey. A screenshot of the survey is provided in Figure 2, while the Supplementary Video S1 is the screen recording of a subject completing the observational experiment.

### 2.4 Interaction experiment

In this experiment, subjects only receive proprioceptive stimuli for making a decision. For the purpose, we let the subject be in touch with the robot tip while performing the movements, as it will be described in the sequel. In order to isolate the proprioception, and hence preserve the purpose of the experiment, the visual and hearing systems are inhibited through a sleep mask and ear muffs.

The interaction experiment is performed with a velocity-controlled UR10 robot, fed with both human and artificial trajectories. Velocity-based control, i.e. the tracking of velocity references, is preferred over position-based control, i.e. the tracking of position references, because a characteristic of human movements is that their tangential velocity profiles can be decomposed into a sequence of submovements with an invariant velocity profile (Morasso and Mussa Ivaldi, 1982; Plamondon and Guerfali, 1998). So, preserving this characteristic should be the key to generating human-like movements with a robot. Since human behavior is expected to be encoded in the velocity information, removing (or reducing) non-desirable effects of control is paramount for this experiment.

In terms of its workspace, the robot performs the handwriting trajectories in its free space (without interacting with surrounding objects) on a virtual horizontal plane. The human participant holds the robot end-effector, specifically designed and 3D-printed to be grasped like a pen. For safety reasons, a physical barrier is placed between the robot and the human, preventing the latter to enter the robot workspace with the entire body. In addition, the robot is programmed to operate close to its workspace boundary, so that its extension towards the human is limited. The end-effector is stiff, so as to prevent introducing artificial dynamics, but also fragile enough, for safety reasons, to break at a collision before provoking injuries to the participant’s hand.

For each participant, the robot executes nine handwriting trajectories (3 Human, 3 MethodA, 3 MethodB) composed as in the previous experiment. To avoid a drop-off in the attention of the participants or the insurgence of fatigue effects, which could affect the experimental results, we reduce the temporal duration of the experiment by executing the three trajectories from the same class one after the other before asking the participant to make a decision. So, each participant interacts with three different sequences made up of three trajectories. The order of the three sequences is randomized among participants in such a way that the human trajectories are presented as the first, the second or the third sequence of movements. After each sequence, the participant is requested to decide if the movements performed by the robot had been generated by a human or by an artificial agent.

Before the experiment, the operator describes the protocol (see the Supplementary Material) and shows to the participant the three movement patterns, printed in the original size and posted on a desk whose top is immediately below and parallel to the virtual plane where the robot movements are executed. This way, the participant is aware of the trajectories’ geometrical features and, during the experiment, can pay attention only to the proprioception of the movement. This should prevent participants from creating a mental image of movements they are experiencing, a phenomenon that could disturb their decision-making process or cause a drop-off in their attention.

A picture of the interaction experiment setup is shown in Figure 2, while the Supplementary Video S2 shows a human subject interacting with the UR10 robot.

### 2.5 Experimental procedure

Each participant was requested to complete the visual test, and a week later, was recalled for the interaction test. The visual test was available on a website for 1 week, and participants were allowed to participate at their convenience. For the interaction test, the participants were admitted one at a time to the room where the experiment was performed to prevent their judgment could be affected by the opinion of other participants.

Through this methodology, the two experiments present a significant difference: while the visual test is performed with no previous experience of the movement patterns (the participants never saw the patterns before taking the test and were completely unaware of the experiment’s purpose), the movements proposed at the interaction experiment were already experienced, although only through observation.

Eventually, and in order to estimate the possible interaction between experiments, we asked the subjects to take the visual test again (with the same trajectories in the same order) immediately after participating to the interaction experiment. The goal is to exclude or confirm that human judgment is somehow affected by experience.

## 3 Results

**FIGURE 3:**
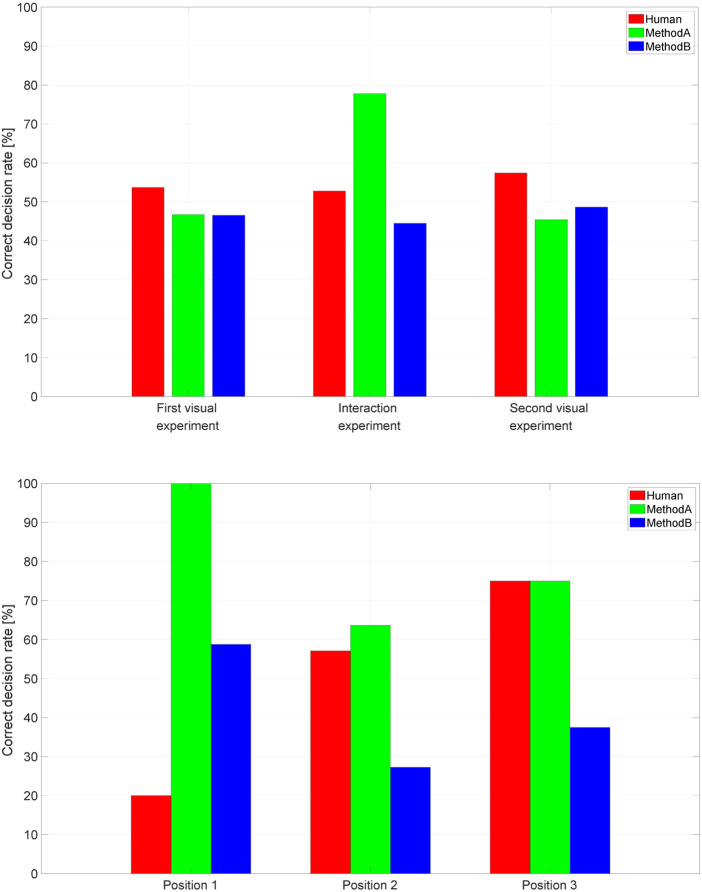
Experimental results over all the performed experiments (top) and accuracy vs. position in the interaction experiment (bottom).

The results of the three experiments, in terms of correct decision percentage, for the three trajectory classes, are summarized in Figure 3 (top).

The results of the first experiment show that there is no evidence that human subjects are able to discriminate human and artificial trajectories by only observing the movement itself, even when their velocity profiles are quite different, as for trajectories generated by MethodA.

The results of the Second experiment reveal some important facts. First, the correct decision rate for MethodA trajectories considerably increases compared to the observation case. This confirms that the proprioceptive sensory system is more sensitive than the visual one in recognizing movements with velocity profiles that are different from those of humans. Second, the correct decision rate of Human and MethodB trajectories in the interaction experiment is similar, thus indicating that MethodB is a better candidate to design human-like trajectories compared to MethodA. As a matter of fact, 55.56% of them are classified as human.

Figure 3 (bottom) reports, for the interaction experiment, the correct decision rate in discriminating human and artificial trajectories depending on the position of the trajectories in the entire sequence. The histogram shows that the later the human trajectories appear in the sequence the higher the correct decision rate. In particular, the correct decision rate for human trajectories is equal to 20%, 57.14%, and 75% when they are the first, second and third of the sequence, respectively. In the case of artificial trajectories, the loss of performance when either MethodA or MethodB trajectories are executed in the second position seems to depend on which trajectory has been executed first in the sequence. Nevertheless, this observation could only be confirmed with experiments involving a larger number of subjects.

These results suggest that the subjects use the first sequence to set a reference, and then evaluate the following ones with respect to it. Moreover, it seems that when evaluating the first sequence, they exhibit some bias towards the artificial category, possibly because they have never experienced a direct interaction with a robot, hence the whole experience is perceived as “artificial”, and such a feeling translates in their judgment. As the experiment proceeds, they adjust to the setting: the bias toward artificial is reduced, they concentrate more on the task and the performance consequently increases.

This interpretation is also confirmed by analyzing the results depending on the number of repetitions of the robot movements the subject asked for before answering the question. In particular, we find out opposite results in the case of human and artificial trajectories, and in particular:

• In the case of human trajectories, the correct decision rate is 57.69% for those executed only once, while it drops to 40.00% for those executed twice;
• In the case of MethodA trajectories, the correct decision rate is 72.41% for those executed only once, and it reaches 100.00% for those executed twice;
• In the case of MethodB trajectories, the correct decision rate is 34.62% for those executed only once, and it reaches 70.00% for those executed twice.

The results of the second visual test, executed soon after the interaction experiment, show no evidence that previous experience of observation and interaction increases the human ability to identify human and artificial movements. Indeed, the results are in line with those of the first one. Furthermore, although participants are able to correctly detect MethodA artificial movements during the interaction experiment, they cannot identify the same movements in the second visual test. This suggests that the difficulty in recognizing human movements, in our experiments, might be unrelated to the lack of experience, while being related to the fact that movement features are more easily perceived through the proprioceptive system than visually.

**FIGURE 4:**
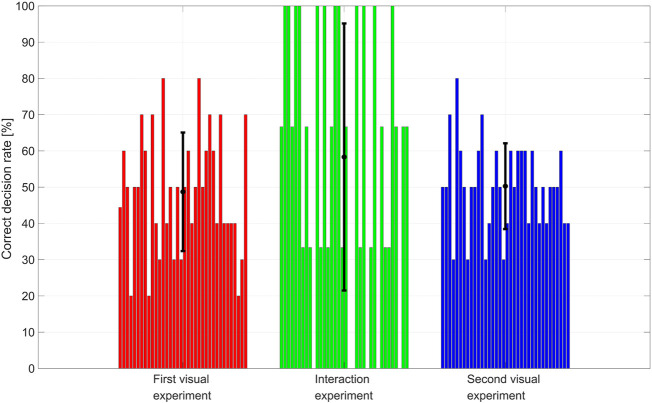
Correct decision rate for each participant and experiment.

The correct decision rate for each participant and experiment is reported in Figure 4. Each histogram, associated with each experiment, has 36 bars, each corresponding to the correct decision rate achieved by each participant in that experiment. Mean and standard deviation over all the participants are overlapped on each histogram. A Wilcoxon test with a significance level of 0.05 is used to compare the results of the two visual experiments. The null hypothesis that the medians of the differences between the two group samples are equal is accepted with *p*-value equal to 0.68.

## 4 Discussion

The robot acceptance by human operators is a complex issue, that is tightly connected to the human perception of motion. In order to assess how different senses perform to this respect, we ran experiments involving human subjects, who were asked to observe and interact with an artificial agent in two different experimental setups, stimulating only one sense at a time. For practicality, the selected movements were extracted from handwriting tasks. In particular, we assessed the validity of the experimental method consisting of exciting the visual system only and, in view of acceptance, compared it with a different experimental method consisting of exciting the proprioceptive system only.

In the first test, the handwriting motion was reproduced in a video, whereas, in the second test, the blindfolded and soundproofed participant was asked to hold the robot end-effector, while being compliant with its motion. In both scenarios, the participants were asked to judge the human likeness of each individual movement, which could embed a human or an artificial time law (derived with one of two different methods from the robotics literature). Eventually, we repeated the first test to assess the influence of experience on judgment.

The outcomes of this research can be summarized as follows:

• a set of participants is not able to reliably distinguish between human and artificial movements shown in a video, even by eliminating visual elements hampering, e.g. a physical robot, and by adding visual elements helping, e.g. a hand with a pen, the self-identification process: whatever class of movements is shown, the correct decision percentage is about 50%;
• during the interaction with a robot executing handwriting movements, participants are able to identify artificial movements adopting uniform velocity (77.78%), but the same does not hold for those that adopt curvature-dependent velocity, which resemble the human ones (44.44%);
• The experience of observation and interaction does not seem to help detect movements in further observation experiments;
• The experience of interaction does seem to help for further interactions, in fact, the correct decision rate for each movement class also depends on the order in which trajectories are executed in the interaction experiment (human movements executed as first are 20.00% correctly identified, while human movements executed as last are 75.00% correctly identified).

From the last observation, a new research question arises: does a human need a comparison to correctly distinguish human and artificial movements? In view of investigating robot acceptance, should the experimental method be defined in terms of “which of these two movements made you feel more comfortable?”. Talking to the participants after they performed the tests, they reported, referring to human movements they were unaware of, that “the movement was too perfect to be performed by a human”. We observed that there are expectations about the robotic/artificial movement that undoubtedly affect the judgment. This also explains why, after gaining some experience of interaction, the correct decision rate tends to increase. Also, in light of these observations, we believe that formulating the question in terms of comfort instead of human likeness is more appropriate in view of robot acceptance. In this case, we would move the focus to the quality of the motion itself, instead of identifying the motion with a robot or a human. This is a research direction that we are willing to investigate in a future work.

In addition, the experimental setup can be improved by including additional tools: for example, a motion tracker could be used to record more generic 3D movements from human subjects, while a 3D simulation environment, featuring a 3D human-like avatar reproducing the motion, could empower the self-identification process and provide further insights about the role and power of observation in the acceptance of artificial movements.

## Data Availability

The original contributions presented in the study are included in the article/Supplementary Material, further inquiries can be directed to the corresponding authors.
