# Supplementary material for: Observation vs. interaction in the recognition of human-like movements
Source: Front Robot AI. 2023 Apr 10;10:1112986. doi: 10.3389/frobt.2023.1112986 (PMC10123277; doi:10.3389/frobt.2023.1112986)
Supplement: Supplementary file 1 [file DataSheet2.pdf]

Interaction experiment – Protocol

The robot will perform a set of movements. The set consists of three writing movements that will be performed horizontally. The set of movements is represented on the sheets placed just below where the movement will be performed. Between each movement, the robot will stop for a few seconds.
For the duration of the set, you must hold the orange handle like a pen. Hold it tightly to prevent slipping.
At the end of the set, answer the following question "Do you believe that the movement performed by the robot was generated by a human or by an artificial agent?". If you cannot provide an answer, you can ask to re-run the motion set.
The robot has been positioned in such a way that it cannot physically reach you, and during the experiment an operator will be constantly present ready to stop the robot in case of anomalies.
When you are ready, we can start the experiment.
For the duration of the experiment, wear the mask over your eyes and the muffs over your ears.
We will repeat this experiment three times for three sets of different movements.
The first set of movements will start in a few seconds.
