# Supplementary material for: Observation vs. interaction in the recognition of human-like movements
Source: Front Robot AI. 2023 Apr 10;10:1112986. doi: 10.3389/frobt.2023.1112986 (PMC10123277; doi:10.3389/frobt.2023.1112986)
Supplement: Supplementary file 3 [file DataSheet1.pdf]

Supplementary Material

1 PARTICIPANTS

	G1	G2
Male	8	27
Female	1	9
Age	23-39	22-29
Mean Age	28.2 (± 5.5)	23.3 (± 1.4)

Table S1. Considered groups of participants: G1 and G2. None of the subjects was included in both G1 and G2. Participants in G1 collected human handwriting trajectories, whereas people in G2 joined the observation and interaction experiments.

2 METHODB TRAJECTORIES

MethodB trajectories respond to a time-optimal planning paradigm. In particular, given a geometric path (or pattern), the time-optimal planning algorithm (Slotine and Yang, 1989) finds a time law that minimizes the trajectory duration, subject to constraints. For a robot, such constraints include maximum torques, velocities, accelerations at the joints, as well as higher order constraints. In addition, the algorithm can accommodate task space constraints, such as maximum velocities and accelerations at the end-effector, that are usually defined by the specific task to be performed.

In our case, the physical robot limits, as reported in the robot's datasheet, allow for much faster movements than the human ones. For this reason, they cannot be used for planning time laws that resemble human ones. Thus, we adopt the classical formulation of time-optimal planning without any robot-dependent constraint and, among the possible constraints that can be defined in the planning instance, we only include Cartesian, i.e. task space, acceleration constraints. The specific acceleration bounds are extracted from the observation of the human profiles, so that the artificial profile naturally takes into account the path curvature and is as close as possible to that of a typical human writing on a sheet of paper. Then, the resulting profiles are normalized so as to match the execution time of the human profile exactly. This choice does not allow the participants to discriminate movements on the basis of their duration.
